# “There Is a Lot of Practice in Not Thinking about That”: Structural, Interpersonal, and Individual-Level Barriers to HIV/STI Prevention among Reservation Based American Indians

**DOI:** 10.3390/ijerph18073566

**Published:** 2021-03-30

**Authors:** Richard F Armenta, Daniel Kellogg, Jessica L Montoya, Rick Romero, Shandiin Armao, Daniel Calac, Tommi L Gaines

**Affiliations:** 1 Department of Kinesiology, California State University, San Marcos, CA 92078, USA; 2 School of Public Health, San Diego State University, San Diego, CA 92182, USA; dkellogg6896@sdsu.edu; 3 Department of Psychiatry, University of California, La Jolla, San Diego, CA 92093, USA; jlmontoya@health.ucsd.edu; 4 Southern California Tribal Health Center, San Diego, CA 92539, USA; rmrjr@yahoo.com (R.R.); sarmao@ucs.edu (S.A.); dcalac66@gmail.com (D.C.); 5 Division of Infectious Diseases and Global Public Health, Department of Medicine, School of Medicine, University of California, La Jolla, CA 92093, USA; togaines@health.ucsd.edu

**Keywords:** HIV/STIs, American Indian, barriers, historical trauma, intergenerational trauma, stigma

## Abstract

American Indians (AI) face significant disparities in HIV/STI morbidity and mortality, and historical, structural, interpersonal, and individual level barriers stymie prevention efforts. The objective of this paper is to examine barriers to HIV/STI prevention among reservation-based AI. We conducted face-to-face qualitative interviews with 17 reservation-based AI community leaders and community members in Southern California on HIV/STI knowledge and attitudes and barriers to prevention. The disruption of traditional coping mechanisms and healing processes were compromised by historical trauma, and this allowed stigmas to exist where they did not exist before. This impacted access to healthcare services and trust in medicine, and is linked to individuals adopting negative coping behaviors that confer risk for HIV/STI transmission (e.g., substance use and sexual behaviors). Most of the participants reported that HIV/STIs were not discussed in their reservation-based communities, and many participants had a misperception of transmission risk. Stigma was also linked to a lack of knowledge and awareness of HIV/STI’s. Limited available services, remoteness of communities, perceived lack of privacy, and low cultural competency among providers further hindered the access and use of HIV/STI prevention services. These findings highlight the need to address the historical, structural, and interpersonal factors impacting individual-level behaviors that can increase HIV/STI transmission among reservation-based AIs. Prevention work should build on community strengths to increase HIV/STI knowledge, reduce stigma, and increase access to preventative care while using culturally grounded methodologies.

## 1. Introduction

At the end of 2018, approximately 1.2 million people were living with HIV in the United States [1], with an estimated 36,400 individuals newly diagnosed that year [1]. Of these new HIV diagnoses, less than 1% were among American Indian and Alaska Natives (AI/AN; [1,2]), who represent 1.7% of the overall U.S. population [3]. Despite having a relatively low number of HIV infections, AI/AN endure a disproportionate burden of HIV-related disparities. From 2010–2017, AI/AN experienced a 39% increase in the annual number of HIV diagnoses with most diagnoses occurring among AI/AN between the ages of 25–44 years and those of male sex [2]. Moreover, approximately 24% of AI/AN with HIV are unaware of their HIV status as compared to 16% of the general U.S. population with undiagnosed HIV [2]. AI/AN are also more likely to die from HIV when compared to other racial/ethnic groups due to later HIV diagnoses and being less likely to receive adequate care [4,5]. One factor contributing to AI/AN HIV-related disparities is high sexually transmitted infection (STI) rates among AI/AN, which can facilitate the acquisition and transmission of HIV [6]. Gonorrhea and chlamydia, for example, are more than four times higher among AI/AN compared to non-Hispanic Whites [7]. Even more worrisome is that surveillance databases likely underestimate HIV/STI rates among AI/AN, due to AI/AN being racially/ethnically misclassified as White and/or Hispanic/Latino [8,9]. STI Disparities among AI/AN raise further concerns given the overlapping risk behaviors that are associated with HIV and STI (e.g., unprotected sex), and thus warrant the need to address HIV risk perception and prevention among AI/AN.

HIV/STI disparities among AI/AN cannot be explained fully by differential involvement in behaviors, such as substance use and risky sexual practices, which increase disease transmission risk [10]. Access to health services and resources, exposure to risky networks, and economic/educational opportunities can directly impact individual health. For example, reservations are usually situated in remote or rural regions with limited accessibility to testing and prevention services. For services that do exist, community members may endure stigma when accessing them or may avoid HIV/STI testing, because they are likely to encounter other community members in local clinics [11]. Historically, AI/AN have faced significant traumas, including massacres, forced removal from land, and banning of cultural practices, and these traumas have contributed to poorer health outcomes in present day AI/AN communities [12]. Historical trauma, coupled with ongoing discrimination and microaggressions, are associated with individual and community health (e.g., mental health, substance use, and violence), including engagement in HIV/STI risk behaviors [11,12].

Communities that are affected by, or at risk for HIV, must be actively involved in prevention efforts to ensure the buy-in and cultural appropriateness of prevention efforts. Community engagement is particularly important in AI/AN communities. Although previous work has identified factors that are related to HIV prevention among AI/AN communities [10,13,14,15,16], the objective of this study was to understand the structural, interpersonal, and individual barriers to HIV/STI prevention among reservation-based AI. A detailed understanding of these barriers will help to guide the selection of effective HIV/STI prevention strategies that meet the needs of reservation-based AI communities in semi-rural or rural areas. 

## 2. Materials and Methods

### 2.1. Study Design and Community Setting 

We conducted a cross-sectional qualitative study using a semi-structured interview guide to capture in-depth information on the barriers and facilitators to HIV/STI prevention among reservation-based AI. The study population involved a multi-tribe consortium served by a Southern California Tribal Health Clinic (SCTHC). This work was conducted in a region that has been ranked as being in the top 10 of all U.S. counties with the highest rates of chlamydia and gonorrhea in 2018 [7]. It is also situated in an area identified as a geographic hotspot for new HIV diagnoses from 2016–2017 [2]. The tribes are located in remote and semi-rural or rural regions of Southern California, which are further characterized by low population density, few public transportation options, and large distances between tribes and the tribal health clinics. Within this context, the community approached a team of AI academic researchers to address HIV/STI awareness and prevention, as this was a topic that had not been addressed within the multi-tribe consortium for over two decades. The lead author and all but one of the authors on this manuscript are AI.

### 2.2. Participants and Sampling Strategy

A total of 17 interviews were conducted with community leaders and community members, at which point saturation of themes was achieved. The participants were 18 years or older who either worked in the community or were a community member. To protect participant and community confidentiality, participant sex and age were not collected in order to limit the amount of identifying information on record. This decision was made in consultation with the tribal IRB and tribal members.

Recruitment was conducted using purposive sampling and targeted outreach methods to recruit diverse perspectives from community members, community stakeholders, tribal leaders, and service providers. The goal was to capture a diversity of HIV/STI attitudes and perceptions across the consortium reservation region. We worked closely with community leaders to ensure that we were obtaining diverse perspectives on HIV/STI in the community from participants who have experience/knowledge of the local health concerns within the community. 

### 2.3. Study Instrument

Qualitative interviews were conducted to obtain information on HIV/STI knowledge and attitudes and barriers to HIV/STI prevention. The semi-structured interview guide was developed in collaboration with community members and leaders, and with feedback from the tribal Institutional Review Board (IRB) to ensure that it was appropriate for use in the local context. The guide included questions, such as: (1) What are the top health concerns in your community? (2) How big of an issue is HIV/AIDS in your community? (3) Where do members of your community find information about HIV and STI? 

### 2.4. Data Collection

Interviews were conducted in a private setting at the health clinic and local community venues. The interviews lasted up to one hour in length, with an average interview length of 41 min. Participants were compensated $20 for their time. Interviews were audio recorded and transcribed by a HIPAA-compliant independent transcription service. Research staff verified the transcription accuracy and removed all personal and community identifying information. 

### 2.5. Ethical Considerations

All of the participants provided written informed consent prior to the start of the interview. The study protocol was approved by the Institutional Review Boards (IRB) at the University of California San Diego (Reference #181535S; Approved: 3 December 2018) and the SCTHC that represents the multi-tribe consortium (Approved: 14 March 2019). This manuscript was also reviewed and approved by the IRB at the SCTHC (Approved: 26 January 2021).

### 2.6. Analysis 

MAXQDA 10 (VERBI, Marburg, Germany) was used to manage qualitative data, coding, and analysis. Two team members conducted a thematic analysis independently and a codebook was developed during the reviewing process of the transcripts. Codes were deductively developed from the topics in the interview guide and inductively developed from additional topics discussed during the interviews [17,18,19]. Codes that were identified early in the process were organized into a codebook and applied to all transcripts. The codes were initially arranged in a hierarchical structure during the first round of coding, organizing information by broad topics (e.g., Health Concerns, HIV & STIs, Barriers to Care). Sub-codes, such as barriers to HIV/STI prevention and treatment, were then formed during the second round of coding (e.g., transportation, lack of privacy, lack of HIV/STI knowledge). The transcripts were recoded when new codes emerged and they were incorporated into the codebook. This allowed for an examination of saturation of codes and, eventually, themes. The saturation of themes was operationalized in relation to both the development of new codes/themes and the degree to which new data repeated what was already expressed in previous data. When discrepancies in coding occurred, differences were resolved through discussions among the research team until consensus was achieved. Four overarching themes emerged during the final phase of analysis, demonstrating the intersecting natures of the codes identified. Demonstrative quotes from participants were selected from blocks of texts coded within the four themes to illustrate the findings. 

## 3. Results

Four overarching themes emerged from the data: (1) Historical/Intergenerational Trauma, (2) HIV/STI Related Stigma, (3) Misperception of Risk, and (4) Privacy/Mistrust in Medicine. The four overarching themes highlighted the historical, structural, interpersonal, and individual level factors impacting HIV/STI prevention with the multi-tribe consortium. Within and between each of the four themes, the identified barriers operated on different social-ecological levels and were largely interrelated, reflecting the complex interplay between historical, structural, interpersonal, and individual factors. For example, many of the barriers stemmed from infrastructure problems that limited knowledge about HIV/STIs, increased stigma, decreased availability of testing and prevention services, and led to a general lack of trust in medical institutions that provide care. 

### 3.1. Historical/Intergenerational Trauma

Community members highlighted the impact of historical and intergenerational trauma on both access to, and engagement in, care and prevention. Many community members discussed the impact of colonization on behaviors and health outcomes.

That comes from a lot of the historical trauma, which they don’t understand—right?—and the intergenerational trauma. Um, and our older family members, um—they’ve just been exhibiting—they’ve just been doing it [dealing with historical trauma] for so long it’s—it’s normalized.

The legacy of historical trauma was described as coping behaviors that are passed down from generation to generation through behavior modeling. Younger community members learn to enact coping behaviors that are modeled by previous generations. Without the stable presence of adults (e.g., parents and grandparents) modeling healthy coping behaviors, many AI/AN youths are vulnerable to adopting negative coping behaviors.

And it just—uh, families, you know, um, teaching their kids what’s right and wrong from wrong. A lot of families—lot of family members grow up, no dad, no mom, so you can kinda tell someone’s—where they’re gonna end up in 10 years, you know. But, uh, it’s—a lot of the tragic stuff does happen to ‘em, so they do go and they get curious, and they go into that, you know, whether it’s be drugs or even sexual activity.

Participants also discussed community members not wanting to deal with historical/intergenerational trauma, including mental health problems which result from this trauma. For example, AI/AN community members may use maladaptive coping behaviors, such as avoidance (e.g., using alcohol and/or drugs to suppress thoughts and memories of trauma), in reaction to trauma that they or their communities have experienced.

But we know when people are not feeling good about themselves or they’re depressed or they’re suicidal or somethin’ like that, you know, they’re—they’re—they’re gonna start making risky behaviors or decisions, and, you know, that, uh, often re—often leads to risky sexual, uh, decisions, so I think that plays a huge role.

In addition to impacting the behaviors of community members, historical/intergenerational trauma was also discussed as causing a disconnect from cultural values and introducing stigma in their communities. Participants discussed how the colonization of tribal communities introduced the concept of shame, which negatively impacted the culture of AI/AN communities.

And, you know, uh, the—the—the boarding schools did a really good job at, you know, bringing on the rest of the shame that we didn’t have, and we didn’t even have a word for shame in our language, you know, prior. So I think that’s a—the—a huge role in where we’re at here, 300 years later, you know.

During discussion of shame as a construct introduced during colonization, AI/AN participants described ways in which the experience of shame led to cultural changes. For example, shame impacted one’s experience and reactions to puberty and the sharing of knowledge regarding sexual practices

Yeah. So like in the traditional culture, uh, you know, when, uh—let’s say for—for a little girl, for instance, when—you know, today when they have their period it’s kinda like a thing—a thing of shame, and—and they often hide it, and they don’t tell their parents, and—and—but in the old culture, you know, they were revered. They were celebrated, and they were taught, you know, um, how to care for themselves, and—and I know they were even taught, you know, uh, by other women, adult women, you know, how to be a good lover, and how to have sex correctly, and—and—and, uh, you know, it wasn’t—it wasn’t a taboo issue.

The participants discussed how various forms of historical trauma (e.g., forceful removal from land and genocide) resulted in significant changes in their communities, including the loss of cultural practices and the introduction of shame. Shame, in turn, led to individuals adopting negative coping behaviors that confer risk for HIV/STI acquisition and prevent the use of HIV/STI prevention services.

### 3.2. HIV/STI Related Stigma

Not only does stigma stymie conversations about HIV/STIs, among other topics, it also impacts community members’ willingness to engage in prevention services, testing, and care.

I think there’s, like, a maybe a stigma with HIV. A lot of people know about it, but they’re not really too familiar about it, or if they have it, you won’t really know, or you’ll hear someone—hey, they might have that, and so, uh, a lot of hearsay. You know, small community, word gets around…

Community members fear both the loss of their anonymity when visiting the tribal clinics and the potential for becoming the focus of hearsay within the community. Some of the participants stated, if a patient is seen receiving services or treatment by someone they know, community members might spread this information.

Uh, gossip talk like that. Um, uh, STIs, I mean, obviously it happens. You know, I mean, uh, j-just awareness, I think, is the biggest thing, a big thing for that, um, yeah.

The hindering effect that stigma has on testing for HIV/STI and seeking services for other stigmatized conditions also leads to a lack of awareness about disease status and prevention methods. This may increase the potential for increased rates of HIV/STIs in the community. The silence from disease stigma facilitates progression to more severe disease within the community, as both the prevention and treatment of disease are under-utilized services.

I don’t think anybody ever feels comfortable because that’s an admission of something that you might have done. Or—or reckless behavior.

### 3.3. Misperception of Risk, Less Knowledge, and Lack of Awareness 

HIV was often discussed as a problem of the past and not a current concern among community members. Most of the participants said that they never knew or heard of someone being infected with HIV, and they perceived that rates of STIs were low in the community. For example, one participant reported being unaware of anyone being infected with HIV or STIs in the community.

I don’t think they’re really aware of the, I mean, I’m sure they are aware of ‘em, but they don’t, I think most people don’t think that they exist here in our communities. Um, it’s not, um, that common to have somebody in the community that’s infected with anything like that, um, as far as like AIDS or HIV. Um, as for the other smaller like, you know, like chlamydia and gonorrhea and stuff like that, you do hear about that every now and then.

Perceptions of low prevalence and risk of HIV/STI appeared to be a factor contributing to the absence of discussion about HIV in the community and the lack of community awareness about HIV/STI prevention services.

Yeah. I think for the community, it’s not on their minds because they don’t hear it, they don’t see it, they haven’t heard HIV/AIDS since the 90s… So, um, some people might be under the impression that it’s been cured.

The participants also stated that community members will not engage in conversations about HIV/STI unless a community member in their social circle becomes infected. Participants do not believe that the community members, especially tribal elders, are comfortable engaging in more in-depth HIV/STI-related discussions due to the stigma and taboo nature that is associated with these topics.

Man, I think that starts in the home. I think, uh—I think the adults are gonna have to be educated better, and—and—and then, you know, carry that on in the home. As that—you know, the-the—those—those kinds of topics, you know, where, uh—where—became taboo through, you know, forced religion and—and stuff like that, and so, you know, the—our older generation, they—they didn’t have the knowledge because it wasn’t somethin’ that was taught to them in boarding school—.

Participants attributed stigma and the lack of educational resources on HIV/STIs as drivers of the silence around HIV risk behaviors, including sexual behaviors and substance use. 

### 3.4. Access to Services, Trust in Medicine, and Privacy as Barriers to HIV/STI Prevention

The participants reported that most people in need of HIV/STI testing and/or treatment would be afraid of going to local clinics due to stigma and fear of questioning from their provider or others at the clinic. This fear is further exacerbated by the rural nature of the consortium communities that are geographically isolated from neighboring communities and have limited access to transportation. The distance and access to transportation creates a barrier for those who need to seek HIV/STI services at local clinics, which can be up to a 45-min. drive for community members. Further, if community members want to attend a clinic outside the community—either because services are not available at the local clinics or because they want to avoid seeing someone they know—the drive is even farther.

There’s a transportation problem, so anything outside of the [local] clinic, because of our population—a lot of it is very rural. There’s not always—there’s—there’s almost no public transportation in those places, and so to get access to those services is—is almost—i- it’s basically impossible.

When people are able to access services, a willingness to engage in care and be tested for HIV/STIs is further hindered by mistrust in medical systems. Medical mistrust has stemmed from historical problems that involve these institutions, particularly problems with the historical mistreatment of AI/AN by medical systems, as well as issues related to privacy. A lack of perceived privacy when seeking services was a prominent barrier to seeking and receiving treatment reported by participants. Participants reported the small community population and interwoven familial relationships make it impossible to maintain anonymity when seeking medical services. The participants reported that they would not seek services or disclose medical information to their provider if their friends or family work at the clinic and/or have access to their medical records. The fear of lack of privacy becomes elevated for those seeking services, especially if the services pertain to stigmatizing issues, including sexual health, substance abuse, or mental health.

don’t trust it, um…and I do see that a lot in the community, um, but they have no other resources to go to, so they have to go to the [local] clinic.

Given that local clinics are often staffed by community members, people are afraid of their information getting out.

But, um, I think they’re reluctant to come in if they know that they’re gonna see somebody they know. So sometimes it’s because of our own—our own families may not get the services that they normally would, because I might be checking you in the front desk. Um, that might be a barrier. Or to get my HIV medication from a cousin who works in the pharmacy.

There were multiple perspectives about privacy and trust from participants.

No, not so much. They don’t trust—trust clinics so much. They—’cause, like, the [local] clinic, you know, everybody knows people, you know? You know? And everybody talks out here. And everybody knows somebody and yeah. So people—people like ch- tend to, like, go away—go out of here somewhere else, you know, to get—for—for services like that.

The participants also emphasized the need for cultural competence (i.e., knowledge about traditional medicine, how culture and health are related, and the history of medical mistreatment) from providers, which was discussed as a barrier to establishing a trusting patient–provider relationship and helping community members to seek care and prevention for HIV/STIs. The participants also discussed the lack of comprehensive care, such that medical professionals may not ask questions related to STI/HIV risk and prevention. A barrier to establishing quality care by tribal service providers stems from the lack of experience and knowledge regarding sensitive or stigmatized health issues in the community. This creates an additional challenge for community members, as they must seek knowledge from multiple sources regarding their health issues.

And—and—and it’s hard to find a lot of Indian people, you know, or, um, minority people that—to—to—to have that education and have that—that—that strength so that you can relate, you know, to—to—to somebody? You know?

These issues are further exacerbated by community members not feeling comfortable talking to clinicians about their health issues due to the perception of high staff turnover and that services are not consistent throughout their visits.

Yeah. Um, there’s like a lot of like people that I know that have, um, struggles with going to the clinic and talking to someone. Um, I’m even one of ‘em that I stopped going…because every time I would go, it was like s—they were s—phasing ‘em out or—you know...

## 4. Discussion

This qualitative study that involved interviews with AI/AN community members and leaders in Southern California provided rich contextual information on barriers to HIV/STI prevention. Historical/intergenerational trauma was described as having broad impacts on the community, including the health of community members. AI reservations are often tight-knight communities given their remoteness and rurality. We found privacy concerns and the ability to maintain confidentially around one’s sexual health to be barriers in accessing prevention services in this setting. The participants also indicated stigma hindered discussion surrounding HIV/STI-related topics in the multi-tribe consortium. Most of the participants reported having never heard about anyone in the community having ever being infected with HIV and, therefore, assuming risk of HIV/STI infection was low. However, national surveillance data show that the region where the reservations are located have some one of the highest rates of chlamydia and gonorrhea in the U.S. [7]. Developing prevention tools is hindered by structural (e.g., limited available services, the rural nature of the communities) and individual (e.g., general lack of knowledge and awareness of HIV/STIs in the community and misperception of risk for disease transmission) level barriers. In addition, the concepts of stigma and shame were explored as important factors impacting the knowledge and awareness of HIV/STIs, as well as individual’s use of available HIV/STI services. Strategies to address issues of stigma are warranted to support the utilization of HIV/STI prevention services.

Limited access to private or public transportation was identified by the participants as a barrier to accessing health services, including HIV/STI prevention. A lack of transportation to distant health care facilities has been previously identified as a prominent barrier to accessing healthcare in other AI/AN communities [20,21]. In small, close-knit, and remote communities, such as those that are involved in the current study, concerns regarding a lack of privacy while accessing health services is a legitimate concern [20,22,23,24]. Recognizing other community members in a healthcare facility serving the AI/AN community is a plausible experience, which raises concerns as to whether an individual might be able to maintain anonymity while obtaining HIV/STI-related health services. The desire for anonymity while obtaining HIV/STI-related health services is likely driven, at least in part, by fear of being subject to HIV/STI-related stigma. Prior studies, for example, indicate that privacy concerns are linked to fear of being subject to discrimination and judgement by others in the context of seeking HIV/STI testing [25,26]. In AI communities, privacy concerns are also exacerbated by a general lack of trust in medical institutions that AI communities often have [27]. Others have found that, even when individuals trust their individual provider, they often maintain anxieties about healthcare systems in general [28]. Limited transportation, privacy concerns, trust in medical institutions, and fear of being subject to HIV/STI-related stigma are likely inter-linked barriers to accessing HIV/STI-related health services. Thus, for HIV/STI prevention efforts to increase their reach among AI/AN communities, consideration of accessibility, the protection of privacy, and addressing institutional trust in medicine is warranted.

Participants discussed multiple types of stigma and shame. HIV/STI related stigma in healthcare settings was discussed as being a barrier to seeking care and engaging in prevention services. Further, stigma from community members and family was described as preventing people from discussing HIV/STIs. The multiple and co-occurring forms of stigma and shame that are described by study participants are situated within a framework where historical traumas (colonization, genocide, forced removal, and loss of culture) provide the context for understanding contemporary stressors (e.g., discrimination, traumatic life events) and the impact that these stressors have on AI health (e.g., HIV, STIs, and mental health) [12,29,30]. Thus, prevention programs need to incorporate how stigma and shame operate at multiple levels to impact health seeking behaviors and engagement in prevention activities within the context of the multiple stressors that impact AI communities.

A lack of knowledge and misperception of personal risk were also identified as barriers to HIV/STI prevention efforts, suggesting a need for culturally tailored HIV/STI education along the life span. Most of the prevention efforts have been directed toward AI/AN adolescents, since they are vulnerable to risky sexual and substance use behavior during their sexual development [31,32,33,34]. However, an emphasis on AI/AN youth could result in missed opportunities, given young to middle age AI/AN adults have been heavily impacted by the HIV epidemic and STIs. From 2010–2017, the rate of HIV diagnoses remained stable for AI/AN age 13–24, but increased by 67% among AI/AN age 25–34 and 60% among AI/AN age 35–44 [2]. Similarly, chlamydia and gonorrhea rates have been the highest for both male and female AI/AN age 20–29 years [7].

Moreover, although our study suggests a need for increased HIV/STI education, other studies have found that increased HIV knowledge among AI/AN does not necessarily lead to decreased HIV risk behaviors [35,36]. Rather, the expected consequences of one’s actions likely motivates HIV/STI risk-related behaviors, such as inconsistent condom use, multiple sexual partners, and substance use [37]. Therefore, our findings highlight the need for multifaceted interventions to address multiple interrelated behaviors and social/sexual contexts influencing HIV/STI risks [38].

HIV/STI prevention efforts may be potentially more impactful and sustainable if they capitalized on the strengths and existing resources of AI/AN communities. Involving tribal communities through ongoing community-based participatory research (CBPR) is one approach to identifying barriers to HIV/STI-related services. CBPR involves the community at all stages of the research process in a collective and reflective process to ensure that community perspectives and knowledge help guide the objective, design, and conduct of the research. CBPR has been shown to be critical in building a sustainable research infrastructure and for implementing strategies to improve health outcomes in AI/AN communities, including efforts to address HIV [39]. The active involvement of the tribal communities in the identification of HIV/STI prevention strategies is intended to help promote the reach of HIV/STI prevention services. CBPR strategies are especially needed to meet the national effort to reduce new HIV infections by 90% through the Ending the Epidemic initiative [40]. Indian Health Service (IHS) provider visit data indicate that men that were aged 20 to 59 years had the highest rate of new HIV diagnoses among people receiving care through IHS [5]. In addition to addressing barriers to accessing HIV/STI-related health service, strategies to reach AI/AN at highest risk for HIV (e.g., men aged 20 to 59) are urgently needed. This is highly important for people who misperceive their risk and do not take preventative action against future HIV/STI infections. Thus, the effective dissemination and implementation of HIV/STI prevention strategies in tribal communities need to adequately consider the “reach” of their strategies. Reach is the absolute number, proportion, and representativeness of individuals who are willing to participate in a service, such as HIV/STI prevention. The active involvement of the tribal communities in the identification of HIV/STI prevention strategies is intended to help promote the reach of HIV/STI prevention services.

This study had a few limitations that should be taken into account when interpreting the results. First, while the saturation of themes was concluded by our team, it is possible we did not capture all perspectives about barriers and facilitators to HIV/STI prevention given the stigma surrounding HIV/STIs that may have inhibited the sharing of information by participants. Given this, our results may not generalize to all rural and semi-rural AI. However, these findings are useful for understanding major barriers to HIV/STI prevention that can be further explored in future studies. Second, we were unable to record participants’ sex, age, or tribal affiliation/location in order to limit the identifiable information and protect participant identity in accordance with tribal IRB. While this information could add additional context to some of our findings, we worked closely with community members and community leaders to recruit a diverse group and capture a variety of perspectives. Future qualitative and quantitative studies with a larger sample size should also collect additional contextual information to confirm and expand on these results as we work to create a community drive prevention program for AI.

## 5. Conclusions

As found in this study, barriers to HIV/STI prevention among AI were rooted in historical and intergenerational trauma that led to increased stigma and shame. The adoption of negative coping behaviors to deal with historical/intergenerational trauma further hindered HIV/STI prevention efforts. Limited services and transportation, remoteness of the communities, privacy concerns, and the lack of HIV/STI knowledge also contributed to reduced engagement in HIV/STI prevention and care. Based on our findings, it is important that culturally grounded HIV/STI prevention programs are developed and delivered by providers who are knowledgeable about the unique challenges that AI may face when seeking HIV/STI care.

## Data Availability

The datasets analyzed during the study are owned by the tribal clinic, which represents the communities from which the data were collected. All requests for data are subject to review and approval by the tribal clinic and principal investigator of the study. Requests should be sent to the corresponding author who will work with the tribal clinic for approval.
